# Social insurance and earnings management: Too rich to be good

**DOI:** 10.3389/fpsyg.2022.934516

**Published:** 2022-10-19

**Authors:** Yunxia Bai, Bofu Zhang

**Affiliations:** School of Economics and Management, Tongji University, Shanghai, China

**Keywords:** social insurance contributions, earnings management, free cash flow, corporate social responsibility, dual collecting system, social security agency, tax bureau, corporate governance

## Abstract

We examine the relationship between social insurance contributions and earnings management for publicly listed firms in China. Our empirical results show that the social insurance contributions burden significantly reduces the degree of earnings management by reducing the level of free cash flow. Additionally, the negative relation between social insurance contributions burden and earnings management is more pronounced when the internal and external social insurance pressures are high and when the firms are large non-state-owned enterprises. We also discuss the heterogeneity among firms for different financing constraints, external financing environment, regional marketization, and internal and external corporate governance. Finally, we further find that under the dual collecting system, although the social security administration is a better collecting agency, the local tax bureau acting with full responsibility is more effective than the collecting system.

## Introduction

China's social security system is constantly evolving (Hussain, 1994). The incidence of social insurance contributions burden is the key issue in the social security policy debate. Most corporate managers think that the burden is too heavy, while many employees regard social insurance contributions as tax-deductible benefits. For the researchers in the field, there is still a gap about how the social insurance contributions burden affects corporate earnings management behaviors. The main reason is the lack of empirical evidence on the causal effect of social insurance contributions burden on earnings management. In this paper, we try to open the black box for understanding China's social security system from the corporate governance perspective with firm-level data from 2008 to 2017. We also provide some recommendations for Chinese social insurance policy makers from the earnings management standpoint.

Social insurance is a transfer program to maintain social stability and promote social equity (Feldstein, 2005). Social insurance is mandatory in China. The social insurance contributions in urban China covers pension insurance, medical insurance, unemployment insurance, work-related injury insurance, and maternity insurance plan. Although China's social insurance system has been developing and improving since its establishment, there are still many practical issues that remained to be solved.

The social insurance contributions in China have attracted particularly high attention from many research areas (Gao et al., 2012; Rickne, 2013; Huang and Han, 2022). In recent years, the central government intends to reduce social insurance rates as a part of tax and fee reduction policy reform. On the other hand, social insurance, as a part of employee protection, provides employees with long-term benefits and wellbeing (Liu et al., 2022). Firms that pay social insurance fees in full amounts are usually in the leading position of corporate governance, employee welfare, and sustainable development. Supported by this background, it is important to explore the impact of social insurance contributions on firms' behaviors and financial decision-making and to provide empirical evidence from the micro perspective for understanding the internal logic of the social insurance system in China.

First, to investigate the effect of social insurance contributions burden on the degree of earnings management, we examined a sample of 18,587 China's firm-year observations over the 10 years from 2008 to 2017. Our empirical results show that the social insurance contributions burden significantly reduces the degree of earnings management. We begin by picturing the trend in the social insurance contributions burden, which is generally upward over the 10 years, and documents a consistent discrepancy between state-owned enterprises (hereafter as SOEs) and non-SOEs. It turns out that the company size matters. The large non-SOEs are among the most affected group under the social insurance contributions burden.

Next, we try to explain the story behind the negative relationship between social insurance contributions burden and earnings management. When the social insurance contributions burden reduces, the firms might obtain abundant free cash flow, and this is especially the case for many publicly listed firms. Jensen (1986) finds that when firms own a large amount of free cash flow, managers tend to waste or over-invest due to conflicts of interest. This raises the problem of agency costs between shareholders and managers generated by cash resources. The problem is particularly severe for firms with poor growth. Any way of forcing managers to give out cash, such as borrowing debts and the distribution of cash dividends can play a binding role. In this paper, we test how social insurance contributions help to reduce agency costs and restrain managers' earning management behaviors based on the free cash flow hypothesis. We select free cash flow as our mediator. The results show that the social insurance contributions burden significantly reduces the level of free cash flow and subsequently lowers the degree of earnings management.

Additionally, the negative relation between social insurance contributions burden and earnings management is more pronounced when the internal and external social insurance pressures are high. Our empirical results are still robust after using different measures, additional control variables, different time intervals, difference-in-differences propensity score matching, and placebo tests.

We also discuss the heterogeneity among firms for different financing constraints, external financing environment, regional marketization, and internal and external corporate governance. The results are still consistent. Finally, we further find that under the dual collecting system, although Social Security Administration acts like a better collecting agency, the local tax bureau with full responsibility is more effective as the collecting system.

To the best of our knowledge, no scholars associate social insurance contributions with earnings management behaviors. This paper attempts to fill up the gap by investigating the impact of social insurance contributions burden on earnings management and revealing its mechanism. Compared with the extant literature, our paper contributes to the literature in three ways. First, it enriches the related literature in the interdisciplinary field of labor and accounting. In recent years, many scholars pay close attention to the effects of social insurance on financial behaviors (Chetty, 2006; Chetty and Looney, 2006; Persson, 2020; Liu et al., 2021). This paper not only enriches the related literature on the micro-economic consequences of social insurance but also generally echoes the research trend in the new field of labor and accounting.

Second, this paper expands the research perspective of earnings management literature. The extant literature mainly examines the determinants of earnings management from the perspective of internal and external corporate governance or macro-economic characteristics, but very few literature works try to explore the impact of labor costs on earnings management (Badertscher, 2011; Dechow et al., 2012). Starting from the angle of social insurance contributions, this paper explores whether and how the social insurance contributions burden affects firms' earnings management behaviors. It reveals the important role of corporate operating cost in earnings management behaviors and provides a deeper understanding of the complex drivers behind earnings management activities.

Third, this paper stresses the prevailing trend of current social security policy reform. This paper empirically finds that the financial pressure caused by the social insurance contributions burden may counteract the managers' earnings management behaviors. Therefore, the reasonable reform policy is of great significance to reduce the agency cost and stimulate the enthusiasm of employees rather than straightly reduce the social insurance contributions. This finding also provides empirical support for the free cash flow hypothesis and provides a greater understanding of the rationality and potential consequences of promoting the universal social insurance collecting system in recent years.

The rest of the paper proceeds as follows: Section Related literature and hypotheses development discusses the related literature and hypothesis development; Section Research design provides data collection and research design; The main empirical results are presented in Section Empirical results; Section Further analysis gives further analyses and Section Conclusion concludes.

## Related literature and hypotheses development

### Related literature

#### Literature on social insurance contributions

The previous research on the economic consequences of social insurance mostly focuses on the macro-level, such as the income distribution effect of social insurance, the impact of social insurance on economic growth, and the impact on national welfare. Acemoglu and Shimer (1999) use the general equilibrium model to find that moderate unemployment insurance not only improves risk sharing but also increases output. Only a few researchers examine the economic consequences of social insurance based on firm-level data. Scholars almost agree that the social insurance contributions burden increases the labor cost of firms, so firms have the incentive to pass on this cost to their employees. Hamaaki and Iwamoto (2010) show that Japanese firms pass on the social insurance contributions burden to their employees. However, there is still controversy about how firms transfer this labor cost, and the related research is mainly tested from the perspective of wage and employment scale. Gruber and Krueger (1991) find that labor protection compensation risks squeeze out workers' wages, but have no significant impact on the number of employees hired by firms. Li and Wu (2013) show that the transfer of social insurance contributions to firms is related to the degree of firm aggregation in the firm aggregation process. Different from the above literature, which uses macro-level social insurance payment data, in recent years, some scholars apply micro-level data to measure the actual social insurance contributions burden. Wei and Xia (2020) use Chinese annual report data and social insurance data of listed firms, respectively. They find that social insurance contributions burden significantly increases the tax avoidance.

#### Literature on free cash flow hypothesis

Several studies provide empirical evidence of the free cash flow hypothesis. To test whether agency cost exists in free cash flow, Griffin (1988) is one of the earliest scholars in this field. Griffin constructs three free cash flow models: neoclassical investment model, pure free cash flow model, and hybrid free cash flow model. After Griffin (1988) and Lang and Litzenberger (1989) examine the correlation between dividend announcement, dividend payment, and free cash flow. To empirically test the effect of the “control hypothesis” on debts, Mann and Sicherman (1991) find that within 2 days before and after the equity issue announcement, the cumulative average prediction error of the stock was −2.64%. At the same time, the cumulative average prediction error of the bond was −0.25% over 2 days before and after the bond issue announcement. The fact implies that shareholders react negatively to equity issue announcements because investors expect managers to misuse any non-bonded funds. This partly reflects the “control hypothesis” effect of debts. Gul (2001) finds that managers of high-debt ratio firms are more willing to choose the last in first out method than managers of low-debt ratio firms because managers of high-debt ratio firms are constrained by external bondholders and are more willing to last in first out to maximize profits. Jaggi and Gul (1999) propose two hypotheses: the debt of low-growth firms is positively correlated with free cash flow (hereafter as FCF) and the positive relation between debt and FCF is more pronounced in large firms. The reason is that high FCF firms need to finance more debts to reduce agency costs, especially when the firm's investment opportunity set is poor. In addition, bond issuing for small firms was hard to achieve due to limited borrowing capacity. In terms of the allocation of cash flow, Guenther et al. (2020) find that firms allocate tax-related cash flow more cautiously than other after-tax cash flow, and suggest that firms invest less and save more tax-related cash flow.

#### Literature on earnings management

On the issue of earnings management related to free cash flow, many scholars have conducted several empirical pieces of research. Christie and Zimmerman (1994) find that managers may mask the decline in corporate value through earnings management, especially for firms with high free cash flows. Gul and Tsui (1997) study the relationship between free cash flow and earnings management from the perspective of audit and considered that managers with high free cash flow and low growth opportunity firms are more likely to manipulate accounting data, so audit would charge higher audit fees. Chung et al. (2005) study the relationship between earnings management, external supervision, and free cash flow. Their empirical results show that there is a significant positive relation between free cash flow and manipulative accrual profits, which indicates that the agency cost of free cash flow is one of the reasons for managers to conduct earnings management. Firms with high growth opportunities are easier to manage earnings than other firms. The higher the free cash flow, the more pronounced the effect.

The literature on the relationship between corporate governance and earnings management can also be divided into macro and micro aspects. At the macroeconomic level, Ball et al. (2003) find that common law countries that pursue corporate governance mechanisms with the goal of profit maximization show higher quality accounting information than civil law countries. Porta et al. (1998) point out that the earnings management behavior of publicly listed firms in different countries is also different due to the different corporate governance mechanisms and legal environments to protect investors. From the micro perspective, the relationship between corporate governance structure and earnings management is empirically studied from three angles: ownership structure, board characteristics, and audit committee characteristics. Demsetz and Lehn (1985) find that equity concentration rate and earnings management is positively correlated. Warfield et al. (1995) believe that executive or institutional investor shareholding can reduce agency costs and, thus, reduce the possibility of management manipulating earnings. Dechow et al. (1996) prove that independent directors can restrain the earnings management behavior of the firm. Beasley (1996) found that firms without financial reporting fraud have a higher proportion of independent directors than firms with fraud. DeFond and Jiambalvo (1991) believe that firms that do not have an internal audit committee provide management with an environment and opportunity to manipulate profits and are more likely to have earnings management. Di Meo et al. (2017) show that management entrenchment is negatively correlated with earnings management and is less detrimental to the firms' value.

### Hypotheses development

To the best of our knowledge, the research on the relation between social insurance and earnings management is absent, but it is important to know the relationship between them since the social insurance contributions burden generates such a great impact on both employers and employees. It also has implications for the ongoing policy reform. Therefore, our first research question investigates the relationship between the social insurance contributions burden and the degree of earnings management. Although the firms have the incentive to pass on social insurance costs to employees by reducing wages or the level of employment, such transfer inevitably leads to deviation from the optimal level of employment and decreases in employee satisfaction, thus, it is hard to achieve. On the other hand, if the social insurance contributions burden is relieved, and the firms are holding an excessive level of free cash flow, the management has the incentive to over-invest and waste the firms' money. Such agency cost inevitably exists in some Chinese listed firms, and subsequently leads to more aggressive earnings management behaviors. Based on these arguments, we propose our first hypothesis as follows:

**H1:** The social insurance contributions burden reduces the degree of earnings management.

Another important problem is discovering the mechanism behind the social insurance contributions burden that affects earnings management behaviors. Social insurance contributions are compulsory, and the law stipulates that workers or their firms should participate in social insurance unconditionally and fulfill their obligation to pay. Thus, it is a mandatory and continuous cash flow expenditure for the firms, which undoubtedly brings greater financial pressure to the firms. Since social insurance is an important part of the labor expenditure for the firms, the cash outflow caused by the social insurance contributions burden eventually reduces the net operating cash flow of the firms. Then the residual cash flow level, after meeting the expected investment level, affects the free cash flow level of the firms. The free cash flow has strategic significance for the firms (Fresard, 2010) because it could provide funding for firms to participate in market competition and maintain market share (Bolton and Scharfstein, 1990). Therefore, in theory, it can be expected that the social insurance contributions burden reduces the level of free cash flow of firms, and then managers have fewer opportunities to conduct earnings management due to the high financial pressure brought by the social insurance contributions burden. That is, the level of free cash flow is the mechanism of earnings management. Many firms are bound to produce a free cash flow surplus in their business activities. Once this part of the cash flow meets the needs of reinvestment, managements have the incentive to make over-investment and on-the-job consumption. This inevitably stimulates and induces more earnings management to cover up misconduct, which deviates from investor objectives and the overall value of the firms. Therefore, how to effectively restrain earnings management is particularly important. Jensen (1986) believes that firms with high growth rates and free cash flow have higher free cash flow agency costs, and management chooses to over-invest to raise salaries and reduce risks. To cover up such behaviors, earnings management is usually carried out to create more profits. Therefore, firms with excess free cash flow conduct more earnings management behaviors. Accordingly, we propose our second hypothesis as follows:

**H2:** The social insurance contributions burden reduces the degree of earnings management by the free cash flow mediator.

The effects of the social insurance contributions burden may also vary according to the internal and external social insurance pressure encountered by the firms. From the viewpoint of internal social insurance pressure, the difficulty of transferring the social insurance contributions burden and the number of employment scales determine the size of the internal social insurance pressure. Labor-intensive firms face greater internal social insurance pressure. The reason is that labor-intensive firms have limited ability to pass social insurance costs to their employees, resulting in increased internal social insurance pressure. After all, it is more difficult for the firm to decide on reducing the number of employees and wages. On the other hand, labor-intensive firms hire more than non-labor-intensive firms, thus generating higher internal social insurance pressure. The most direct consequence of hiring more laborers is higher social insurance expenditure. Because of the above reasons, labor-intensive firms often face higher pressure on social insurance contributions, and ultimately bear greater financial pressure. Thus, management teams have more motivation to ease the heavy burden of social insurance contribution rather than to misconduct the free cash flow.

From the perspective of external social insurance pressure, firms with “empty accounts” or low pension funds' looseness face greater external social insurance pressure. It is well-known that in many parts of China, there exist “uncollected” pension accounts. The severe deficit of social insurance funds in these areas is filled by local government, which undoubtedly brings great pressure to local government financing and might have a permanent impact on local government behavior. To ensure the income of local pension funds, local governments need to strengthen their intervention in firms within their jurisdiction and require firms to sign market-oriented contracts with employees in accordance with the labor contract law. It may require greater local government intervention in areas where pensions are “empty” or where pension looseness is low. The social insurance collection in these regions needs to be more stringent. In this case, it is more difficult for firms to evade the arrears of social insurance contributions and must pay social insurance in full amounts on time. Subsequently, the management must obey strict regulations and is less likely to over-invest or misconduct, resulting in fewer earnings management behavior. Therefore, the internal and external social insurance pressure enhances the negative relation between social insurance contributions burden and earnings management to a certain extent. Accordingly, we propose the third hypothesis as follows:

**H3:** The negative relation between social insurance contributions burden and earnings management is more pronounced when the internal and external social insurance pressure is high.

Besides, we also examine several other possible explanations for the effect of social insurance contribution on earnings management, including changes in ownership structure, changing firm characteristics, dual collecting system, and some other factors. We also conduct several sets of additional analyses and robustness tests.

## Research design

### Sample and data collection

The sample of this study contains 2008–2017 Chinese A-share publicly listed companies. The reason for choosing 2008 as the starting year of the sample is because Chinese government officials have implemented the new “Accounting Standards” since 2007. The new standards require listed companies to disclose social insurance contribution costs in their financial statements, and this study gives a 1-year buffer period for the adjustment. In the notes to the financial statements of listed companies, we can inquire about the opening balance, the increase in the current period, the decrease in the current period, and the closing balance of the “payroll payable” and their detailed information. Thus, the social insurance costs disclosed by various listed companies are not standardized, so the paper adjusts the number to ensure that all the social insurance cost data are accurate.

To ensure the validity of the data, we screen the sample data based on the following criteria. First, we exclude the financial services and special treatment (ST) firms. The reason we exclude these firms since these firms have very different financial structures and performances on the balance sheet. Second, we eliminate the sample with abnormal and missing data for key variables. Third, we merge the processed data with the discretionary accruals data. Also, to control the extreme values with the risk of data overflow, the continuous variables are winsorized on 1 and 99% percentile. Finally, we obtain 15,772 observations over the 10-year-period from 2008 to 2017. The financial data for this paper are from CSMAR data Library and WIND database; the degree of relaxation of regional pension funds (*LOOSE*) data are from the past years of China's Labor Statistics Yearbook.

### Variable definitions

The social insurance contributions burden (*SIC*) is defined as the ratio of current social insurance contributions over the revenue to measure the social insurance contributions burden (i.e., *SIC* = social insurance contributions/revenue). Specifically, this paper measures the social insurance contributions burden by dividing the current increase in “social insurance expense” under the subject of “payroll payable” over the current revenue. The greater the ratio, the heavier the social insurance contributions burden borne by the listed companies. By taking the revenue as the denominator, we treat the social insurance contributions as an important component of the firms' labor costs. The standardization of the revenue can comprehensively depict the social insurance contributions burden for the business activities of the firm.

This paper uses two types of discretionary accrual as the measure of the degree of earnings management. We use Jones's (1991) model to measure the discretionary accrual from the change of revenue and the change of fixed assets. The model is as follows:


(1) $$\begin{array}{l} \begin{array}{lll} \frac{{\text{TA}}_{i,t}}{A_{i,t-1}} & = & \alpha_{1}(\frac{1}{A_{i,t-1}})+\alpha_{2}(\frac{\Delta REV_{i,t}}{A_{i,t-1}}) \end{array}\\ \;\begin{array}{ll} + & \alpha_{3}(\frac{\Delta PPE_{i,t}}{A_{i,t-1}})+\varepsilon_{i,t} \end{array} \end{array}$$

*A*_*i, t*−1_ represents the total assets of the last period; Δ*REV*_*i, t*_ is the increment of revenue; and *CAPITAL*_*i, t*_ is the fixed assets. The residual error calculated by the above equation is the discretionary accruals.

The modified Jones model (Dechow et al., 1995) is based on the Jones model by adding the change of accounts receivable. The specific model is as follows:


(2) $$\begin{array}{l} \begin{array}{lll} \frac{{\text{TA}}_{i,t}}{A_{i,t-1}} & = & \alpha_{1}(\frac{1}{A_{i,t-1}})+\alpha_{2}(\frac{\Delta REV_{i,t}-\Delta REV_{i,t}}{A_{i,t-1}}) \end{array}\\ \;\begin{array}{ll} + & \alpha_{3}(\frac{\Delta PPE_{i,t}}{A_{i,t-1}})+\varepsilon_{i,t} \end{array} \end{array}$$

The level of free cash flow refers to Wei and Xia (2020). This paper uses the net operating cash after the standardization of total assets plus the difference between expected investment and sustainable investment to measure the level of free cash flow within the firms. Specifically, this paper uses the regression model of Richardson (2006) to estimate the expected investment level of the firm:


(3) Invi,t=β0+β1Invi,t−1+β1Sizei,t−1                +β1Levi,t−1+β1Cashi,t−1                +β1Agei,t−1+β1Qi,t−1+β1Returni,t−1+∑​Year+∑​Industry+εi,t

For the above equation, *Inv*_*i, t*_ is the firm's new investment, which is equal to the difference between the cash paid for the purchase of fixed assets, intangible assets, and other long-term assets in the current year and the cash recovered from the disposal of fixed assets, intangible assets, and other long-term assets divided by the total assets at the beginning of the year; *Size*_*i, t*−1_ represents the natural logarithm of total assets at the beginning of the year; *Lev*_*i, t*−1_ indicates the level of total liabilities at the beginning of the year; *Cash*_*i, t*−1_ indicates cash holdings at the beginning of the year; *Age*_*i, t*−1_ represents the natural logarithm of the number of years from the firms' listing to the t-1 year; *Q*_*i, t*−1_ indicates the Tobin *Q*-value at the beginning of the year; and *Return*_*i, t*−1_ represents the firms' annual stock return for the t-1 year; the model also controls both industry and year fixed effect. Based on the above regression model, this paper can predict the expected investment level of *i* firm in *t* year (exp_inv). Sustaining investment is equal to the sum of depreciation of fixed assets and amortization of intangible assets divided by total assets. The level of free cash flow (*FCF*) is equal to the difference between the operating net cash flow after the standardization of total assets and the expected investment and sustaining investment of the firm. The greater the value, the higher the level of free cash flow within the firm.

The control variables selected in this baseline model include firm size (*SIZE*), debt level (*LEV*), return on assets (*ROA*), firms' capital intensity (*CAPITAL*), the ratio of largest shareholder shareholding (*FIRST*), the ratio of institutional investor shareholding (*INSTITU*), and equity structure (*STATE*). In addition, this paper also controls the year and industry fixed effects. Table 1 reports the specific definitions of the main variables in this paper.

**Table 1:** Definitions of main variable.

**Variable name**	**Symbol**	**Description**
Discretionary accrual	*DA*	Jones (1991) or modified Dechow et al., 1995
Social insurance contributions burden	*SIC*	Change of social insurance contributions in current period/revenue
Free cash flow	*FCF*	Net cash flows from operating activities / (total assets – expected investment – sustaining investments)
Regional pension looseness	*LOOSENESS*	Local pension balance/number of pension recipients
Labor density	*INTENSIVE*	Natural logarithm of Number of employees / fixed assets
firm size	*SIZE*	Natural logarithm of total assets
Financial leverage	*LEV*	Total liabilities / total assets
Return on total assets	*ROA*	Net profit / total assets
Corporate capital intensity	*CAPITAL*	Fixed assets / total assets
% of shares held by the largest shareholders	*FIRST*	Number of shares held by the largest shareholder / total shares
% of shares held by Institution	*INSTITU*	Number of shares held by institutions /total shares
Capital structure	*STATE*	When a listed firm belongs to a state-owned enterprise, take 1, otherwise take 0
Year fixed effect	*YEAR*	Using 2008 as the base year, nine dummy variables are set
Industry fixed effect	*INDUSTRY*	A total of 54 industry dummy variables are created based on the CSRC Industry Classification Standard, 2012. Manufacturing industry is partitioned according to the secondary industry.

This table presents the main variables used in this paper. DA is our dependent variable. It comes from either Jones' model (DA) or modified Jones' model (MODDA). Our variable of interest is social insurance contributions burden SIC, which is the ratio of social insurance contributions over revenue. Free cash flow FCF is used as our mediator. Every other variable is used either as a control variable or as the variable under control.

Source: The authors' calculations are based on data from the sources indicated in Section Sample and data collection (a merge from hand collecting, CSMAR, and WIND).

### Empirical model

Next, we set up the empirical models for the social insurance contributions burden and earnings management. To test the relationship between social insurance contributions burden and earnings management, this paper constructs the baseline regression model:


(4) $$\begin{array}{l} {\left|\text{DA}\right|}_{i,t}=\beta_{0}+\beta_{1}SIC_{i,t}+{\sum \beta_{j}\times Control}_{i,t}+\varepsilon_{i,t} \end{array}$$

*DA* is the degree of earnings management; *SIC* is the social insurance contributions burden; *Control* is the control variable, and the ε is the residual term. When the regression coefficient β_1_ of *SIC* is significantly negative, it shows that the social insurance contributions burden significantly decreases the degree of earnings management, which means that Hypothesis 1 holds. At the same time, this paper also examines the differences between the social insurance contributions burden and earnings management in different sample sub-groups between internal and external social insurance pressure. If the negative effect of the social insurance contribution burden on earnings management is more significant in the sample sub-group with greater internal or external social insurance pressure, Hypothesis 3 is supported empirically.

For testing the mechanism behind the social insurance contribution burden that affects earnings management, this paper uses the mediating effect test which has been widely used in the field of corporate finance in recent years to conduct empirical analysis. This paper tests the mediating effect through the following steps:


(5) $$\begin{array}{lll} fcf_{i,t} & = & \beta_{0}+\beta_{1}SIC_{i,t}+{\sum \beta_{j}\times Control}_{i,t}+\varepsilon_{i,t} \end{array}$$

(6) $$\begin{array}{l} \begin{array}{lll} |DA{|}_{i,t} & = & \beta_{0}+\beta_{1}SIC_{i,t}+\beta_{2}fcf_{i,t} \end{array}\\ \;\begin{array}{ll} + & \sum^{\text{​}}\beta_{j}\times Control_{i,t}+\varepsilon_{i,t} \end{array} \end{array}$$

For the equations above, *fcf*_*i, t*_ represents the free cash flow level of the firm *i* in year *t*. We first use Equation 5 to test the relationship between the social insurance contributions burden and the level of free cash flow. If the social insurance contributions burden coefficient β_1_ of the regression result is significantly negative, it shows that the social insurance contributions burden significantly reduces the level of free cash flow. Next, we use the social insurance contributions burden (*SIC*) and the free cash flow level (*FCF*) as independent variables for the regressions that involve the degree of earnings management in Equation 6. If the regression coefficient of β_1_ is no longer significant compared with that of Equation 4, then the level of free cash flow is the mediating mechanism that the social insurance contributions burden affects earnings management, implying that Hypothesis 2 holds empirically. Noting the consistence with the practice of relevant literature, this paper clusters the standard errors of the regression model at the firm level.

## Empirical results

### Descriptive statistics

The descriptive statistics of the main variables in this paper are shown in Table 2A. For the social insurance contributions burden (*SIC*), the mean is about 1.47%, which means that on average, the firms spend roughly 1.47% on social insurance contributions relative to revenue. Generally, the A-share listed firms are the elites among all Chinese firms. Thus, the social insurance contributions burden is reasonably low. The mean of discretionary accrual (*DA*) is 0.0063 and the median is 0.0008. This shows that overall, the listed companies have positive discretionary accrual, in other words, most listed firms manipulate their earnings upward.

**Table 2A:** Descriptive statistics of major variables.

**Var**	**Mean**	**S.D**.	**Median**	**Min**	**Max**	**Observations**
*DA*	0.0669	0.0759	0.0445	0.0006	0.4598	18,587
*MODDA*	0.0698	0.0802	0.0455	0.0007	0.4869	18,587
*SIC*	0.0147	0.0124	0.0115	0.0006	0.0705	18,531
*FCF*	−0.0352	0.0747	−0.0344	−0.2364	0.1737	15,920
*INTENSIVE*	234.5766	416.5739	100.5256	4.0659	2775	18,551
*LOOSENESS*	5.4160	4.2155	4.3850	0.7633	17.6148	18,411
*SIZE*	22.0635	1.2735	21.8869	19.5033	25.9356	18,587
*LEV*	0.4309	0.2046	0.4284	0.0460	0.8639	18,587
*ROA*	0.0510	0.0402	0.0419	0.0015	0.2063	18,587
*CAPITAL*	0.2278	0.1705	0.1920	0.0022	0.7221	18,587
*FIRST*	0.3607	0.1514	0.3433	0.0908	0.7573	18,237
*INSTITU*	0.3893	0.2354	0.3946	0.0010	0.8728	18,587

This table presents the descriptive analysis of the main variables used in our study. The sample covers the period 2008–2017, and all observations are subject to the criteria described in Section Empirical results. We take the absolute value of discretionary accruals from both Jones' model (DA) and modified Jones' model (MODDA). Some variables contain missing values, which drop from our dataset.

Source: The author's calculations are based on data from the sources indicated in Section Sample and data collection [merged from hand collecting, Wei and Xia (2020), CSMAR, and WIND].

The level of free cash flow (*FCF*) has a mean of −0.035, which indicates that there is a cash gap in the internal cash flow on average. The average labor density (*INTENSIVE*) is 234.58, but the standard deviation is as high as 416.57, which shows that the labor intensity of different firms varies greatly. The average pension looseness (*LOOSENESS*) is 5.42, which is not high. It is equivalent to giving the accumulated balance of pension to the population in need of pension, with an average of 54,200 yuan per person. There is a great difference in pension looseness among different provinces based on the standard deviation and max/min value. The descriptive statistics of other variables are very close to the extant literature.

Table 2B presents the correlation matrix for various variables. Discretionary accruals (DA) are negatively correlated with *SIC, FCF, INTENSIVE, LOOSENESS, SIZE, CAPITAL*, and *INSTITU* at the 1% statistically significant level. *DA* is positively and significantly correlated with *LEV, ROA*, and *FIRST*.

**Table 2B:** Correlation matrix.

**Variables**	**(1)**	**(2)**	**(3)**	**(4)**	**(5)**	**(6)**	**(7)**	**(8)**	**(9)**	**(10)**	**(11)**
(1) *DA*	1.000										
(2) *SIC*	−0.104^***^	1.000									
(3) *FCF*	−0.056^***^	−0.016^**^	1.000								
(4) *INTENSIVE*	−0.067^***^	0.070^***^	0.045^***^	1.000							
(5) *LOOSENESS*	−0.054^***^	−0.025^***^	0.026^***^	0.049^***^	1.000						
(6) *SIZE*	−0.044^***^	−0.143^***^	0.033^***^	0.612^***^	0.069^***^	1.000					
(7) *LEV*	0.101^***^	−0.206^***^	−0.086^***^	0.249^***^	−0.089^***^	0.492^***^	1.000				
(8) *ROA*	0.103^***^	−0.032^***^	0.309^***^	−0.002	0.014^*^	−0.104^***^	−0.383^***^	1.000			
(9) *CAPITAL*	−0.172^***^	0.097^***^	−0.094^***^	0.124^***^	−0.161^***^	0.074^***^	0.069^***^	−0.092^***^	1.000		
(10) *FIRST*	0.016^**^	0.003	0.031^***^	0.171^***^	−0.040^***^	0.226^***^	0.089^***^	0.070^***^	0.080^***^	1.000	
(11) *INSTITU*	−0.046^***^	0.014^*^	0.127^***^	0.261^***^	0.006	0.416^***^	0.193^***^	0.098^***^	0.071^***^	0.303^***^	1.000

All variables are defined in Table 1 and all the continuous variables are winterized at 1 and 99% to mitigate the effect of outliers.

#x0002A;**: p < 0.01,

** p < 0.05,

* p < 0.1.

**Figure 1:**
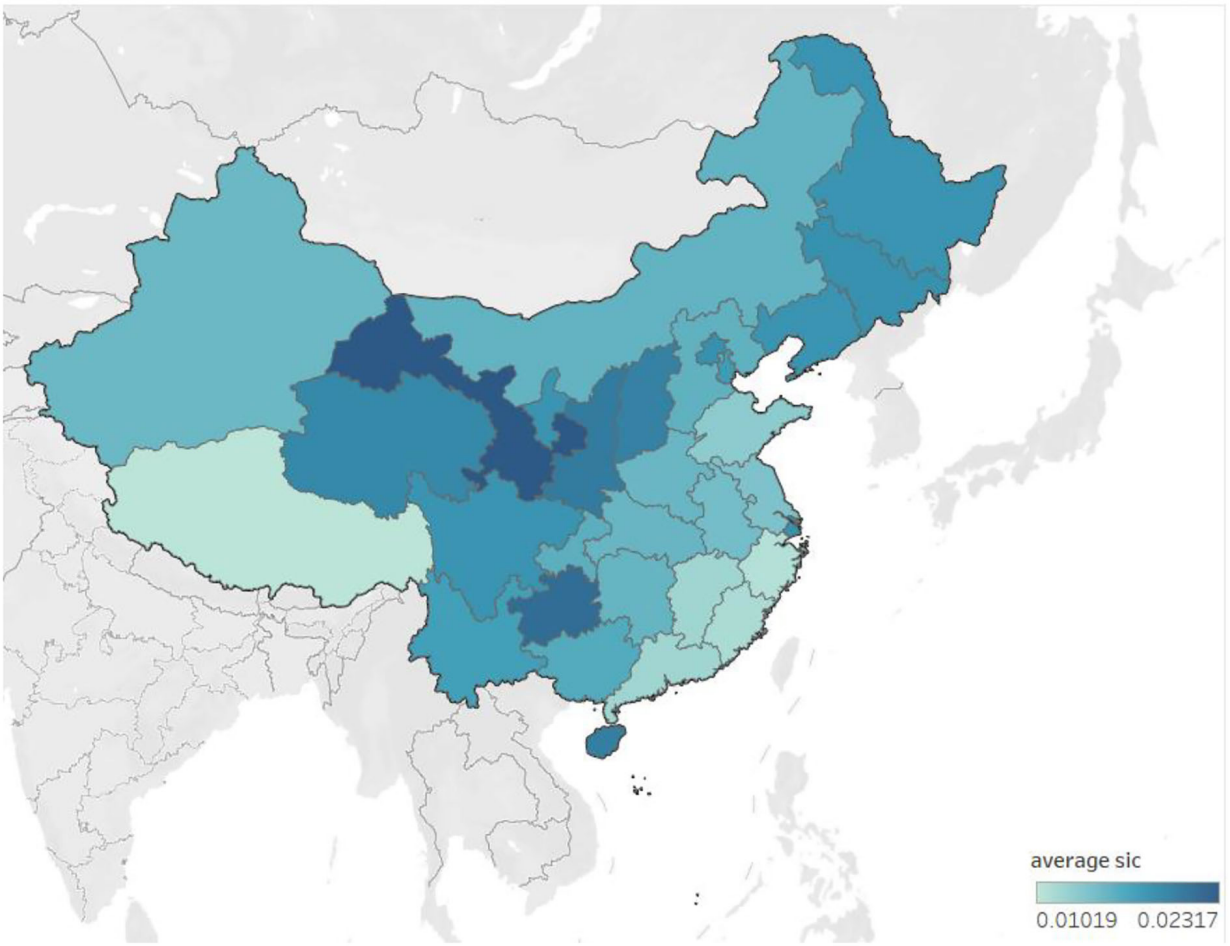
Social insurance contribution burden for A-share listed firms. This figure shows the social insurance contributions burden for A-share listed firms across Mainland China (Taiwan is part of China but does not have A-share listed firms as of 31 December 2017). It plots the average social insurance contributions burden per province over 10 years from 2008 to 2017. The dark color indicates a heavy burden on average for listed firms located in that province, while the light color shows the opposite. Source: The author's calculations are based on data from the sources indicated in Section Sample and data collection.

Figure 1 reports the level of social insurance contributions burden across China. In general, Figure 1 shows that the social insurance contributions burden in the economically developed areas is lower than in economically underdeveloped areas. The social insurance contributions burdens in the province of Guangdong, Zhejiang, Fujian, Jiangsu, and other coastal economically developed areas are lower due to more active private enterprises or more intense labor competition in these areas. Beijing and Shanghai are two special cases, which have a heavier burden than the surrounding cities because the two regions are usually the headquarters for national enterprises.

### Regression analysis

We now examine the relationship between the social insurance contributions burden and earnings management (Hypothesis 1). Table 3 tests the main regression on whether the social insurance contributions burden decreases the degree of earnings management. Table 3 shows that the regression coefficient of the social insurance contributions burden (*SIC*) is −0.1845, which is significantly negative at the 1% level, indicating that the social insurance contributions burden significantly decreases the degree of earnings management, thus, Hypothesis 1 is supported empirically. At the same time, the result of the regression is also significant in an economic sense, that is, for each unit increase of the social insurance contributions burden, the degree of earnings management decreases by about 0.1845 on average. The finding of this regression implies that the heavy social insurance contributions burden brings heavy financial pressure to firms, which may force firms to take various measures to deal with it. Earnings management seems to have become an important way for firms to “neutralize” the social insurance contributions burden. In this regard, this paper also provides further empirical evidence in the following mechanism tests to examine whether the social insurance contributions burden, as an important expenditure of firms, reduces the free cash flow level of listed companies and whether this leads the management team to adopt less radical earnings management strategies.

**Table 3:** Social insurance contributions and earnings management.

**Panel A**		
	**(1)**	**(2)**
	***DA***	***MODDA***
*SIC*	−0.1845^***^	−0.1912^***^
	(−2.950)	(−2.976)
*SIZE*	−0.0060^***^	−0.0062^***^
	(−7.582)	(−7.623)
*LEV*	0.0635^***^	0.0643^***^
	(12.804)	(12.633)
*ROA*	0.3008^***^	0.3082^***^
	(13.675)	(13.569)
*CAPITAL*	−0.0554^***^	−0.0563^***^
	(−10.603)	(−10.634)
*FIRST*	0.0092^*^	0.0101^**^
	(1.869)	(1.988)
*INSTITU*	−0.0118^***^	−0.0127^***^
	(−3.723)	(−3.862)
*STATE*	−0.0034^**^	−0.0034^**^
	(−2.120)	(−2.066)
*Intercept*	0.2075^***^	0.2176^***^
	(11.651)	(12.038)
Industry	Yes	Yes
Year	Yes	Yes
*N*	18,184	18,184
*R^2^*	0.1272	0.1256

All standard errors are clustered by firm. The t-statistics are reported in parentheses below the coefficient estimates.

***, **, and * represent statistically significant at the 1, 5, and 10% levels, respectively.

This table presents the baseline regression results in our study for both Jones' model [specification (1) DA] and modified Jones' model [specification (2) MODDA]. Coefficients measure the effect of social insurance contributions burden on the discretionary accruals. Our regressor of interest is the social insurance contributions burden (SIC), which is the ratio of social insurance contributions over revenue. All specifications include year and industrial dummy, which are omitted in this table.

Source: The author's calculations are based on data from the sources indicated in Section Sample and data collection (merged sample data from hand collection, CSMAR, and WIND).

**Figure 2:**
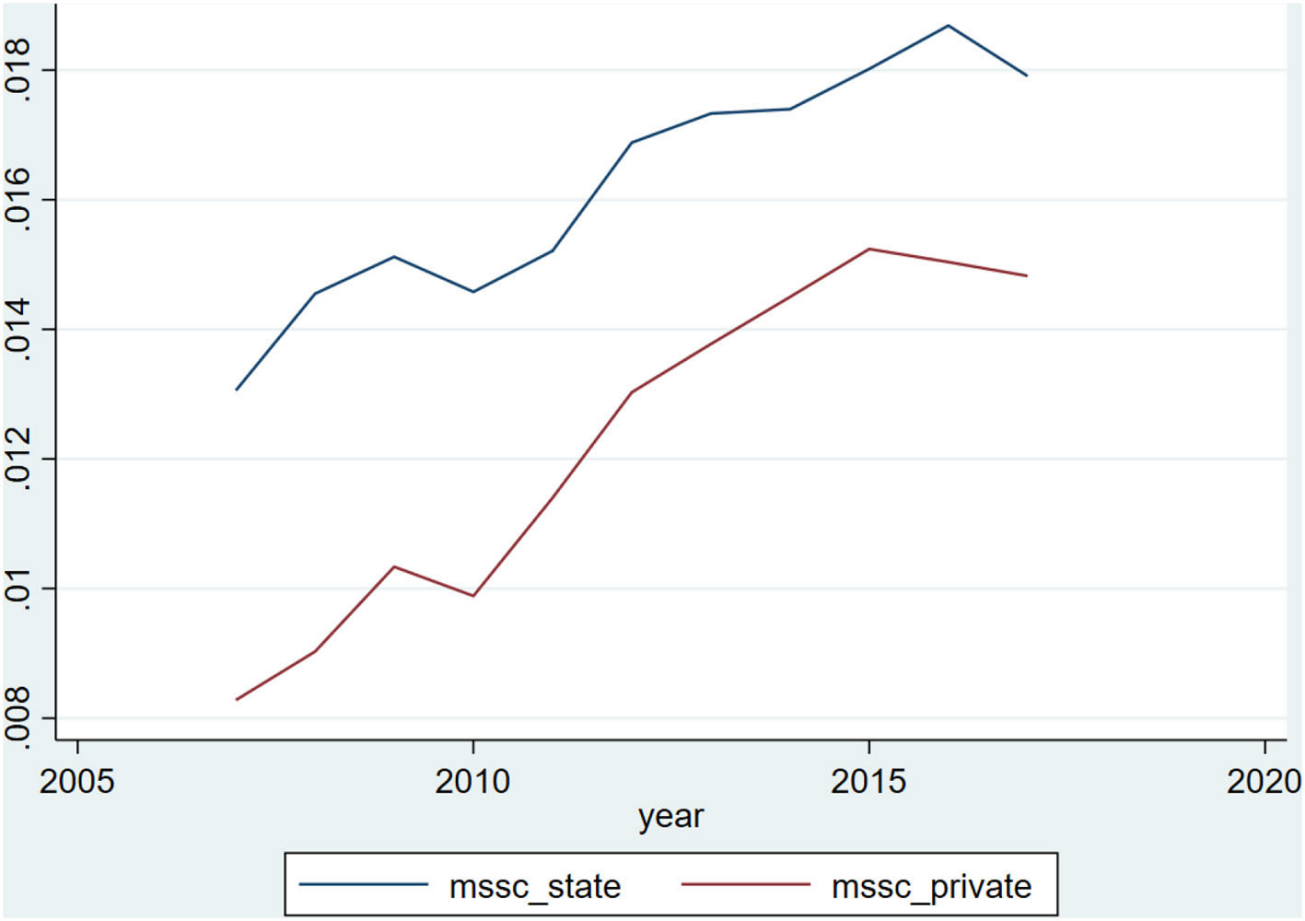
Social insurance contributions burden SOE vs. NON-SOE. Mean annual social insurance contributions burden over the sample period separately for state-owned enterprises and non-state-owned enterprises. This graph plots the annual mean SSC over the sample period, 2008–2017. SSC is the ratio of social insurance contributions to revenue. In this graph, a firm is an SOE if its controlling shareholder belongs to the state, otherwise, it is a non-SOE. All observations are subject to the criteria described in Table 1. Source: The author's calculations are based on data from the sources indicated in Section Sample and data collection.

We follow Dyreng et al.'s (2017) model to examine heterogeneity in firms. Figure 2 illustrates the visual evidence for our first hypothesis by plotting the average social insurance contributions burden independently for state-owned enterprises (SOEs) and non-SOEs. Two immediate results are evident in Figure 2. First, state-owned enterprises on average have a higher social insurance contributions burden than non-state-owned enterprises every year during our sample period. Thus, the result suggests that the average SOE has a much higher social insurance contributions burden than a non-SOE.

Second, both SOE and non-SOE exhibit a declining social insurance contributions burden over time at approximately the same rate. At the beginning of the period in 2008, the average SOE has an *SIC* of about 1.3% and the average non-SOE had an *SCI* of ~0.82%. By the end of the sample period, SOEs raise to an average of 1.8%, while non-SOEs contribute roughly 1.4%. This result seems to highly contradict the government's intention to reduce the social insurance contributions burden, while both SOEs and non-SOEs bear an increasingly high social insurance contributions burden.

In Panel B of Table 3, we present the result from estimating our baseline model for SOEs and non-SOEs separately with discretionary accrual for both Jones and modified Jones' model. The coefficients of *SIC* are negative and statistically significant for both SOEs and non-SOEs. But the negative effect of social insurance contributions burden on the degree of earning management is greater for SOEs than for non-SOEs. The closing gap between SOEs and non-SOEs as the burden increased in recent years has been shown in Figure 2. Also, the intercept for SOEs is higher and statistically significant for both Jones and modified Jones models. This indicates that initially, SOEs have a higher degree of earnings management than non-SOEs.

**Table 3:** SOE vs. non-SOE.

**Panel B**				
	**(1)**	**(2)**	**(3)**	**(4)**
	***DA_SOE***	***DA_NONSOE***	***MODDA_SOE***	***MODDA_NONSOE***
*SIC*	−0.2177^***^	−0.1556^*^	−0.2132^**^	−0.1711^*^
	(−2.597)	(−1.694)	(−2.477)	(−1.813)
*SIZE*	−0.0055^***^	−0.0067^***^	−0.0056^***^	−0.0069^***^
	(−5.395)	(−5.341)	(−5.375)	(−5.330)
*LEV*	0.0599^***^	0.0664^***^	0.0627^***^	0.0658^***^
	(8.855)	(9.154)	(8.909)	(8.797)
*ROA*	0.3311^***^	0.2857^***^	0.3379^***^	0.2967^***^
	(9.837)	(9.803)	(9.610)	(9.815)
*CAPITAL*	−0.0442^***^	−0.0658^***^	−0.0453^***^	−0.0665^***^
	(−5.844)	(−8.528)	(−5.948)	(−8.335)
*FIRST*	0.0146^**^	0.0080	0.0178^**^	0.0075
	(1.964)	(1.204)	(2.270)	(1.091)
*INSTITU*	−0.0115^**^	−0.0158^***^	−0.0124^**^	−0.0169^***^
	(−2.181)	(−3.879)	(−2.250)	(−4.032)
*Intercept*	0.2027^***^	0.2294^***^	0.2077^***^	0.2430^***^
	(8.224)	(8.223)	(8.371)	(8.446)
Industry	Yes	Yes	Yes	Yes
Year	Yes	Yes	Yes	Yes
*N*	8,119	10,065	8,119	10,065
*R^2^*	0.1347	0.1311	0.1321	0.1300

All standard errors are clustered by firm. The t-statistics are reported in parentheses below the coefficient estimates.

***, **, and * represent statistically significant at the 1, 5, and 10% levels, respectively.

This table presents the regression results in our study for both Jones' model and modified Jones' model. Specifications (1) DA_SOE and (3) MODDA_SOE are for state-owned enterprise sub-samples. Specifications (2) DA_Non and (4) MODDA_Non are for non-state-owned enterprise sub-samples. Coefficients measure the effect of the social insurance contributions burden (SIC) on the discretionary accruals for different sub-samples. Our regressor of interest is SIC, which is the ratio of social insurance contributions over revenue. All specifications include year and industrial dummy, which are omitted in this table.

Source: The author's calculations are based on data from the sources indicated in Section Sample and data collection.

One reason the effect of the SOEs and non-SOEs are different could be due to the significant difference among firm sizes. The SOEs normally have a much larger firm size relative to their counterpart. Next, we try to test whether the effect of social insurance contributions burden on earnings management differs by firm size. Thus, we try to examine this in two steps. First, we try to examine whether large firms significantly differ from small firms regardless of the nature of property rights. Second, we test whether SOEs differ from non-SOEs of the same size. Panel C of Table 3 shows convincing evidence that the increase in social insurance contributions burden has a greater reducing impact on earnings management for large firms and for large non-SOEs. This is mainly because large firms enjoy less financial constraint and have more free cash to spend, and large non-SOEs have the least internal control over managers' usage of free cash flow. Thus, the increase in social insurance contributions burden causes the greatest impact on those large non-SOEs.

**Table 3:** Firm size.

**Panel C**				
	**(1)**	**(2)**	**(3)**	**(4)**
	***DA_LARGE***	***DA_SMALL***	***DA_NONSOE_LARGE***	***DA_SOE***
*SIC*	−0.1912^**^	−0.1187	−0.3455^***^	−0.1354
	(−2.157)	(−1.237)	(−2.894)	(−1.598)
*LEV*	0.0498^***^	0.0649^***^	0.0492^***^	0.0473^***^
	(7.208)	(9.426)	(5.400)	(7.500)
*ROA*	0.3117^***^	0.3316^***^	0.2999^***^	0.3220^***^
	(9.300)	(10.579)	(7.399)	(9.477)
*CAPITAL*	−0.0411^***^	−0.0717^***^	−0.0531^***^	−0.0452^***^
	(−5.774)	(−9.199)	(−4.779)	(−5.995)
*FIRST*	0.0200^***^	0.0010	0.0274^***^	0.0083
	(2.817)	(0.136)	(2.882)	(1.086)
*INSTITU*	−0.0168^***^	−0.0105^**^	−0.0239^***^	−0.0167^***^
	(−3.646)	(−2.311)	(−3.907)	(−3.234)
*STATE*	−0.0048^**^	−0.0051^*^		
	(−2.289)	(−1.922)		
*Intercept*	0.0809^***^	0.0907^***^	0.0782^***^	0.0980^***^
	(6.637)	(7.607)	(5.988)	(7.010)
Industry	Yes	Yes	Yes	Yes
Year	Yes	Yes	Yes	Yes
*N*	9,249	8,935	5,186	8,119
*R^2^*	0.1298	0.1289	0.1216	0.1289

All standard errors are clustered by firm. The t-statistics are reported in parentheses below the coefficient estimates.

***, **, and * represent statistically significant at the 1, 5, and 10% levels, respectively.

This table presents the regression results in our study for only Jones' model. Specification (1) DA_LARGE is for large enterprise sub-samples. Specification (2) DA_SMALL is for small enterprise sub-samples. Specification (3) DA_NONSOE_LARGE is for large non-state-owned enterprise sub-samples. Specification (4) DA_SOE is for general state-owned enterprise sub-samples. Coefficients measure the effect of the social insurance contributions burden (SIC) on the discretionary accruals for different sub-samples. Our regressor of interest is SIC, which is the ratio of social insurance contributions over revenue. All specifications include year and industrial dummy, which are omitted in this table.

Source: The author's calculations are based on data from the sources indicated in Section Sample and data collection.

### Mediating effects

Next, we examine which mediator affects the relationship between the social insurance contributions burden and earnings management. Table 4 empirically tests Hypothesis 2, whether free cash flow is the mediating mechanism through which the social insurance contributions burden affects earnings management. Table 4 column (1) shows that the social insurance contribution burden (*SIC*) significantly reduces the free cash flow at the 5% level. The above findings confirm the conventional view that social insurance contributions are paying at a high proportion, causing heavy financial pressure on firms. Table 4 column (2) considers both the social insurance contributions burden and free cash flow level in the regression of earnings management to explain variables. The regression coefficient of the free cash flow level (*FCF*) is significantly negative at the 1% level, which indicates that the tighter the free cash flow, the higher the degree of earnings management. In general, the above results support the free cash flow as a mediator, that is, the social insurance contributions burden decreases the degree of earnings management by reducing the level of free cash flow.

**Table 4:** Social insurance payment burden, free cash flow level, and earnings management—mediation effect.

	**(1)**	**(2)**	**(3)**	**(4)**	**(5)**
	***FCF***	***DA***	***MODDA***	***SGMEDDA***	***SGMEDMODDA***
*SIC*	−0.2244^***^	−0.1216^**^	−0.1086^*^	−0.1283^***^	−0.1162^**^
	(−3.619)	(−1.980)	(−1.709)	(−2.580)	(−2.275)
*SIZE*	−0.0032^***^	−0.0057^***^	−0.0058^***^	−0.0056^***^	−0.0057^***^
	(−4.093)	(−7.126)	(−7.103)	(−9.678)	(−9.570)
*LEV*	0.0071	0.0482^***^	0.0485^***^	0.0477^***^	0.0480^***^
	(1.466)	(10.020)	(9.905)	(12.784)	(12.522)
*ROA*	0.5957^***^	0.2759^***^	0.2796^***^	0.2719^***^	0.2758^***^
	(26.908)	(11.597)	(11.400)	(16.428)	(16.218)
*CAPITAL*	0.0096^*^	−0.0457^***^	−0.0457^***^	−0.0454^***^	−0.0453^***^
	(1.807)	(−8.581)	(−8.517)	(−11.340)	(−11.003)
*FIRST*	−0.0127^**^	0.0075	0.0083^*^	0.0076^*^	0.0083^**^
	(−2.523)	(1.541)	(1.650)	(1.946)	(2.054)
*INSTITU*	0.0168^***^	−0.0065^**^	−0.0068^**^	−0.0067^**^	−0.0069^**^
	(5.223)	(−2.097)	(−2.123)	(−2.425)	(−2.439)
*STATE*	0.0170^***^	−0.0003	−0.0002	−0.0003	−0.0002
	(9.856)	(−0.206)	(−0.148)	(−0.232)	(−0.187)
*FCF*		−0.0985^***^	−0.1021^***^	−0.0990^***^	−0.1026^***^
		(−6.667)	(−6.756)	(−12.580)	(−12.685)
*Intercept*	−0.0375^**^	0.1899^***^	0.1975^***^	0.1895^***^	0.1969^***^
	(−2.152)	(10.555)	(10.802)	(14.937)	(15.107)
Industry	Yes	Yes	Yes	Yes	Yes
Year	Yes	Yes	Yes	Yes	Yes
*N*	15,772	15,772	15,772	15,772	15,772
*R^2^*	0.1959	0.1379	0.1371	0.1739	0.1801
*F*				52.8679	55.1405

All standard errors are clustered by firm. The t-statistics are reported in parentheses below the coefficient estimates.

***, **, and * represent statistically significant at the 1, 5, and 10% levels, respectively.

This table presents the mediating effect results in our study for both Jones' and modified Jones' models. Specification (1) uses FCF as a mediator. Specifications (2) and (3) are the results for mediating effects. Specifications (4) and (5) are the Sobel–Goodman tests to examine whether a mediator carries the influence of an independent variable to a dependent variable. Coefficients measure the effect of social insurance contributions burden on the discretionary accruals for different groups. Our variable of interest is social insurance contributions burden SIC, which is the ratio of social insurance contributions over revenue. All specifications include year and industrial dummy, which are omitted in this table.

Source: The author's calculations are based on data from the sources indicated in Section Sample and data collection.

The above empirical results show that the regression coefficient of social insurance contribution burdens (*SIC*) is lower than that of Table 3 (from −0.28 to −0.24). To ensure the validity of our conclusion, we use the Sobel-Goodman tests to test whether a mediator carries the impact of the social insurance contributions burden on earnings management in the last row of column (2) of Table 4. The *F*-value of the Sobel-Goodman test is significant at the level of 1%, which means the mediation effect has passed the significance test. This means that free cash flow plays a role as a mediator in the relationship between social insurance contributions burden and earnings management. In other words, the social insurance contributions burden causes the decline of firms' free cash flow level. As a result, firms must decrease earnings management in response to the decline of the free cash flow level. One potential explanation is: that reducing the social insurance contributions rate frees up more cash flow to spend and invest without the need for manipulating earnings. Thus, Hypothesis 2 holds.

### Internal and external social insurance payment pressure

The relationship between the social insurance contributions burden and earnings management might be affected by internal and external social insurance pressure. Tables 5A,B further examines the impact of social insurance contributions burden on earnings management under different internal and external social insurance pressures. As mentioned earlier, this paper uses labor density to measure the internal social insurance pressure and uses local pension looseness to measure the external social insurance pressure. Tables 5A,B shows that the social insurance contributions burden (*SIC*) is significantly positive in the high labor density group, while the level of significance decreases in the low labor density group. This shows that compared with the low labor density group, the negative effect of social insurance contributions burden on earnings management is more pronounced within the high labor density group. The reason might be that the higher the labor density, the greater the social insurance pressure for the firm, and the firms need to bear greater social insurance expenses for their employees, thus, the firms have less funding to manipulate and fewer investment projects to consider, bringing less opportunity for earnings management.

**Table 5A:** Internal and external social insurance pressure.

	**Panel A: Internal**		**Panel B: External**	
	**Low intensity**	**High intensity**	**Low looseness**	**High looseness**
	**(1)**	**(2)**	**(3)**	**(4)**
	***DA***	***DA***	***DA***	***DA***
*SIC*	−0.0146	−0.2023^***^	−0.1627^*^	−0.2102^**^
	(−0.137)	(−2.784)	(−1.876)	(−2.248)
*CONTROLS*	Yes	Yes	Yes	Yes
Industry	Yes	Yes	Yes	Yes
Year	Yes	Yes	Yes	Yes
*Intercept*	0.1901^***^	0.1368^***^	0.1586^***^	0.2343^***^
	(5.435)	(5.452)	(5.864)	(9.853)
Chow's test	0.0000^***^		0.0081^***^
*N*	8,921	9,263	8,185	9,999
*R^2^*	0.1362	0.1269	0.1332	0.1300

All standard errors are clustered by firm. The t-statistics are reported in parentheses below the coefficient estimates.

***, **, and * represent statistically significant at the 1, 5, and 10% levels, respectively.

This table presents the regression results in our study for Jones' model. Specification (1) DA reports the results for low–intensity internal social insurance pressure. Specification (2) DA reports the results for high–intensity internal social insurance pressure. Specification (3) DA reports the results for low looseness of external social insurance pressure. Specification (4) DA reports the results for high looseness of external social insurance pressure. Our variable of interest is social insurance contributions burden SIC, which is the ratio of social insurance contributions over revenue. All specifications include year and industrial dummy, which are omitted in this table.

Source: The author's calculations are based on data from the sources indicated in Section Sample and data collection.

**Table 5B:** Internal and external social insurance pressure.

	**Panel A: Internal**		**Panel B: External**	
	**Low intensity**	**High intensity**	**Low looseness**	**High Looseness**
	**(1)**	**(2)**	**(3)**	**(4)**
	***MODDA***	***MODDA***	***MODDA***	***MODDA***
*SIC*	−0.0347	−0.1901^**^	−0.1662^*^	−0.2191^**^
	(−0.319)	(−2.516)	(−1.902)	(−2.219)
*CONTROLS*	Yes	Yes	Yes	Yes
Industry	Yes	Yes	Yes	Yes
Year	Yes	Yes	Yes	Yes
*Intercept*	0.2021^***^	0.1463^***^	0.1713^***^	0.2400^***^
	(5.586)	(5.687)	(6.182)	(9.963)
Chow's test	0.0000^***^		0.0108^**^	
*N*	8,921	9,263	8,185	9,999
*R^2^*	0.1339	0.1265	0.1353	0.1250

All standard errors are clustered by firm. The t-statistics are reported in parentheses below the coefficient estimates.

***, **, and * represent statistically significant at the 1, 5, and 10% levels, respectively.

This table presents the regression results in our study for modified Jones' model. Specification (1) MODDA reports the results for low–intensity internal social insurance pressure. Specification (2) MODDA reports the results for high–intensity internal social insurance pressure. Specification (3) MODDA reports the results for low looseness of external social insurance pressure. Specification (4) MODDA reports the results for high looseness of external social insurance pressure. Our variable of interest is social insurance contributions burden SIC, which is the ratio of social insurance contributions over revenue. All specifications include year and industrial dummy, which are omitted in this table.

Source: The author's calculations are based on data from the sources indicated in Section Sample and data collection.

Tables 5A,B also shows that the social insurance contributions burden (*SIC*) is significantly negative in the high pension looseness group but relatively less significant in the low pension looseness group. Chow's test is statistically significant at the 1% level. It further verifies that the difference in coefficients between sub-samples is statistically significant. Chow's test is valid since we assume that most control variables do not statistically differ for sub-samples. This means that the negative effect of social insurance contributions burden on earnings management is more pronounced in the high pension looseness group than in the low pension looseness group. This seems to contradict our original hypothesis that lower pension looseness results in decreasing earnings management, but it is indeed that the high looseness group has greater external financial pressure. The possible explanation is that the higher the local pension looseness, the greater the local pension balance target due to strict collecting from the social insurance agency or local tax bureau, causing greater external social insurance collecting pressure on the firms. Thus, the managers must bear greater financial pressure and have limited interest in over-investment or other tunneling behaviors, and subsequently conduct fewer earnings management.

Above all, the internal and external social insurance pressure does strengthen the negative relation between social insurance contributions burden and earnings management. In other words, Hypothesis 3 is supported empirically.

### Robustness tests

We conduct our robustness tests in various ways. First, as previously shown, we use both Jones and modified Jones models to measure earnings management for most of our regression results.

Second, we exclude the competitive interpretation by filling in additional control variables. Due to the strong correlation between social insurance contributions burden and wage level, the results in this paper might also be caused by the change in wage level. To exclude this competitive interpretation, we add the firms' wage level (*WAGE*, the total credit amount payable to employees divided by the number of employees) as the control variable in the regression model. The empirical results are reported in Table 6 column (1). Table 6 column (1) shows that, after controlling the wage level, the social insurance contributions burden still generates a regression coefficient that is still significantly positive at the 5% level. This means that after considering the impact of the wage-level change, the conclusion is still valid.

**Table 6:** Controlling fixed effect at the corporate level and intensity of regional tax bureau.

	**(1)**	**(2)**
	***DA***	***MODDA***
*SIC*	−0.2301^**^	−0.2400^**^
	(−2.335)	(−2.358)
*FCF*	−0.0801^***^	−0.0840^***^
	(−8.846)	(−8.982)
*SIZE*	0.0008	0.0009
	(0.531)	(0.541)
*LEV*	0.0603^***^	0.0605^***^
	(9.167)	(8.910)
*ROA*	0.4242^***^	0.4343^***^
	(18.844)	(18.679)
*CAPITAL*	−0.0628^***^	−0.0658^***^
	(−8.075)	(−8.181)
*FIRST*	0.0180^*^	0.0253^**^
	(1.828)	(2.484)
*INSTITU*	−0.0048	−0.0056
	(−1.181)	(−1.344)
*STATE*	−0.0094^**^	−0.0088^*^
	(−1.964)	(−1.774)
*Intercept*	0.0386	0.0423
	(1.161)	(1.230)
Industry	Yes	Yes
Year	Yes	Yes
*N*	15,774	15,774
*R^2^*	−0.0952	−0.0942
*F*	57.7528	58.4897

All standard errors are clustered by firm. The t-statistics are reported in parentheses below the coefficient estimates.

***, **, and * represent statistically significant at the 1%, 5%, and 10% levels, respectively.

This table conducts the robustness tests in our study for both Jones' and modified Jones' models. Specification (1) DA and (2) MODDA reports fixed effect models. Coefficients measure the effect of social insurance contributions burden on the discretionary accruals for different groups. Our variable of interest is social insurance contributions burden SIC, which is the ratio of social insurance contributions over revenue. All specifications include year and industrial dummy, which are omitted in this table.

Source: The author's calculations are based on data from the sources indicated in Section Sample and data collection.

Third, to eliminate various sample noises, Table 7A excludes the competitive interpretation; Table 7B exploits the 2012 labor protection policy change and shows whether the effect is more pronounced for firms with high social insurance contributions. The year 2012 is indeed a special year for the social security system in China. A controversial labor policy in China was implemented by the government in 2012 that strengthens labor protection. Specifically, we run the regression before 2012 and after 2012 for both low and high (below or above the mean value) corporate social responsibility (*CSR*), respectively. The corporate social responsibility measures come from the WIND database.

**Table 7A:** Exclusion of competitive interpretation and sample replacement interval.

	**(1)**	**(2)**	**(3)**	**(4)**
	***DA1***	***MODDA1***	***DA2***	***MODDA2***
*SIC*	−0.1990^***^	−0.2074^***^	−0.0585	−0.1901^**^
	(−3.148)	(−3.195)	(−0.522)	(−2.516)
*SIZE*	−0.0062^***^	−0.0063^***^	−0.0060^***^	−0.0036^***^
	(−7.566)	(−7.565)	(−4.678)	(−3.103)
*LEV*	0.0638^***^	0.0647^***^	0.0741^***^	0.0649^***^
	(12.747)	(12.579)	(9.569)	(8.948)
*ROA*	0.2936^***^	0.3011^***^	0.3551^***^	0.3359^***^
	(13.300)	(13.173)	(10.229)	(10.340)
*CAPITAL*	−0.0550^***^	−0.0561^***^	−0.0690^***^	−0.0443^***^
	(−10.438)	(−10.504)	(−8.297)	(−6.154)
*FIRST*	0.0092^*^	0.0103^**^	0.0183^**^	0.0232^***^
	(1.863)	(1.996)	(2.474)	(3.267)
*INSTITU*	−0.0119^***^	−0.0129^***^	−0.0112^**^	−0.0125^***^
	(−3.758)	(−3.922)	(−2.270)	(−2.767)
*STATE*	−0.0036^**^	−0.0036^**^	−0.0075^***^	−0.0027
	(−2.226)	(−2.151)	(−2.963)	(−1.323)
*WAGE*	0.0020	0.0019		
	(1.340)	(1.247)		
*Intercept*	0.1907^***^	0.2013^***^	0.2078^***^	0.1463^***^
	(8.834)	(9.132)	(7.127)	(5.687)
Industry	Yes	Yes	Yes	Yes
Year	Yes	Yes	Yes	Yes
*N*	18,055	18,055	7,711	9,263
*R^2^*	0.1259	0.1244	0.1638	0.1265

All standard errors are clustered by firm. The t-statistics are reported in parentheses below the coefficient estimates.

***, **, and * represent statistically significant at the 1, 5, and 10% levels, respectively.

This table conducts the robustness tests in our study for both Jones' and modified Jones' modes. Specifications (1) DA and (2) MODDA are the results after taking additional noisy variables (WAGE) into account. Specification (3) is the sample before 2013 and (4) is the sample after 2013 (including 2013). Coefficients measure the effect of social insurance contributions burden on the discretionary accruals for different groups. Our variable of interest is social insurance contributions burden SIC, which is the ratio of social insurance contributions over revenue. All specifications include year and industrial dummy, which are omitted in this table.

Source: The author's calculations are based on data from the sources indicated in Section Sample and data collection.

**Table 7B:** 2012 labor protection policy change.

	**(1)**	**(2)**	**(3)**	**(4)**
	**DA_low** **CSR_before**	**DA_high** **CSR_before**	**DA_low** **CSR_after**	**DA_high** **CSR_after**
*SIC*	0.2641	−0.1674	−0.3257^***^	−0.2558^**^
	(1.164)	(−1.082)	(−3.973)	(−2.038)
*CONTROLS*	Yes	Yes	Yes	Yes
Year	Yes	Yes	Yes	Yes
Industry	Yes	Yes	Yes	Yes
Chow test			2.07^***^	
*N*	1,989	3,936	6,774	3,699
*R^2^*	0.1817	0.2116	0.0802	0.0788

All standard errors are clustered by firm. The t-statistics are reported in parentheses below the coefficient estimates.

***, **, and * represent statistically significant at the 1, 5, and 10% levels, respectively.

This table presents the regression results in our study for Jones' model. Specification (1) DA_lowCSR_before and (2) DA_lowCSR_before are for low and high CSR before 2012 labor protection policy respectively. Specification (3) DA_lowCSR_after and (4) DA_lowCSR_after are for low and high CSR after 2012 labor protection policy, respectively. Coefficients measure the effect of the social insurance contributions burden (SIC) on the discretionary accruals for different sub-samples. Our primary interest is SIC, which is the ratio of social insurance contributions over revenue. All specifications include controls, year, and industry dummy, which are omitted in this table.

Table 7B show that before 2012, the coefficients of *SIC* for both low and high *CSR* groups are not statically significant. After 2012, the coefficients of *SIC* for both low and high *CSR* groups are statistically significant. This means that the labor protection policy is functioning and impact the effect of social insurance contributions on earnings management. Furthermore, the Chow test shows the distinction between low and high *CSR* is significant. Thus, after 2012, social insurance contributions reduce earnings management by a larger scale for low *CSR* firms compared with high *CSR* firms. This implies that after the labor protection policy, high *CSR* firms are more likely to follow the guideline. So, the social insurance contributions function less to alleviate the earnings management problem. Comparatively, those low *CSR* firms still rely on passive indicators such as social insurance contributions to reduce earnings management. In other words, the labor protection policy works but works better for good firms.

Fourth, we use the difference-in-differences propensity score matching (PSM-DID) approach to deal with selection bias in Table 8. In 2016, the Ministry of Human Resources and Social insurance and the Ministry of Finance jointly issued the *Notice on the Periodic Reduction of Social Insurance Rates*, which requires that, from 1 May 2016, the social insurance contribution paid by firms' employees in some regions should be reduced. The social insurance contributions rates in Beijing, Shanghai, Tianjin, Sichuan, Chongqing, Anhui, Jiangxi, Xinjiang, Shanxi, Henan, Hubei, Guangxi, Guizhou, Hunan, Gansu, and Ningxia were affected by this policy. By using this policy shock, the paper tries to investigate the effects of exogenous shock on earnings management behavior by constructing PSM-DID. Specifically, this paper treats the areas that reduce the proportion of pension insurance contributions as the treatment group, and the rest of the areas as the control group. First, this paper uses the propensity score matching method to pair a total of 5,980 nearest neighbor matching samples based on their debt level, return on assets, firms' capital intensity, the ratio of largest shareholder shareholding, institutional investor shareholding ratio, and growth. After matching the above variables, the difference among the variables between the treated group and the control group is <3%. Additionally, the mean value of each variable between the treatment group and the control group is not pronounced at the 5% significant level, which indicated that the matching process is well-executed. After matching the propensity score, this paper uses the paired samples to conduct the difference-in-differences test. We set the treatment group as a dummy (*TREAT*). For the firms located in the treated group areas, *TREAT* takes the value of one, otherwise zero. We also set the policy implementation time dummy variable (*POLICY*), which indicates that the year after the policy implementation (2016 or after) is set to be one. Finally, we construct the interactive term *POLICY_TREAT* to investigate the impact of the promulgation of the notice on earnings management. Table 8 shows that the *POLICY_TREAT* regression coefficient is significantly negative at the 10% level. This shows that after the exogenous impact of the policy, the proportion of social insurance contributions in the regions where the firms are located decreases, and the degree of earnings management of the firms also decreases significantly. This implies that exogenous shocks are not only useful for identifying the causal relationship between the social insurance contributions burden and earnings management but also support the previous research that the social insurance contributions burden decreases earnings management behaviors.

**Table 8:** Difference–in–differences propensity score matching (PSM–DID).

	**(1)**	**(2)**
	***DA***	***MODDA***
*POLICY_TREAT*	0.0068^*^	0.0069^*^
	(1.803)	(1.796)
*POLICY2*	−0.0387^***^	−0.0433^***^
	(−6.852)	(−7.268)
*TREAT2*	−0.0029	−0.0028
	(−1.199)	(−1.137)
*CONTROLS*	Yes	Yes
*Intercept*	0.0768^***^	0.0816^***^
	(6.783)	(7.038)
Industry	Yes	Yes
Year	Yes	Yes
*N*	5,980	5,980
*R^2^*	0.1210	0.1205

All standard errors are clustered by firm. The t-statistics are reported in parentheses below the coefficient estimates.

***, **, and * represent statistically significant at the 1, 5, and 10% levels, respectively.

This table presents the PSM-DID robustness check in our study for both Jones' and modified Jones' models. Specification (1) DA and (2) MODDA reports the results for PSM-DID. Coefficients measure the effect of social insurance contributions burden on the discretionary accruals for different groups. Our variable of interest is social insurance contributions burden SIC, which is the ratio of social insurance contributions over revenue. All specifications include year and industrial dummy, which are omitted in this table.

Source: The author's calculations are based on data from the sources indicated in Section Sample and data collection.

Fifth, to verify the reliability of the difference-in-difference model estimation conclusion, a series of placebo tests were carried out by constructing a pseudo-year policy implementation year and random sampling in Table 9. The placebo test is based on a pseudo-policy year. We construct the counter-factual test by changing the policy implementation point (assuming that the policy is implemented 1, 2, 3, or 4 years ahead of schedule, respectively). If the coefficient of the interaction term in the regression results is still significant, it implies that the social insurance reduction policy is not the reason for the social insurance contributions burden to significantly reduce earnings management. It might be caused by some other policies or random factors. If the interaction term is no longer significant, this indicates that the policy does have an impact. The test results are shown in the table. The estimated coefficients of the interaction terms in columns (2–5) of Table 9 are not significant, and this reversely indicates that the results obtained above are not due to other policy and random factors.

**Table 9:** Placebo test—pseudo year policy.

	**(1)**	**(2)**	**(3)**	**(4)**	**(5)**
	***RIGHT***	***−1***	***−2***	***−3***	***−4***
*POLICY_TREAT*	0.0068^*^	0.0037	0.0057	0.0015	0.0001
	(1.802)	(0.996)	(1.534)	(0.378)	(0.022)
*POLICY1*	−0.0394^***^	−0.0379^***^	−0.0389^***^	−0.0368^***^	−0.0362^***^
	(−6.928)	(−6.648)	(−6.822)	(−6.384)	(−6.215)
*TREAT2*	−0.0024	−0.0020	−0.0034	−0.0015	−0.0007
	(−1.018)	(−0.764)	(−1.155)	(−0.455)	(−0.164)
*LEV*	0.0563^***^	0.0563^***^	0.0563^***^	0.0563^***^	0.0563^***^
	(8.447)	(8.442)	(8.443)	(8.439)	(8.441)
*ROA*	0.2511^***^	0.2507^***^	0.2510^***^	0.2501^***^	0.2499^***^
	(7.493)	(7.491)	(7.499)	(7.473)	(7.465)
*CAPITAL*	−0.0565^***^	−0.0565^***^	−0.0564^***^	−0.0565^***^	−0.0566^***^
	(−6.766)	(−6.769)	(−6.767)	(−6.777)	(−6.778)
*FIRST*	0.0164^*^	0.0163^*^	0.0163^*^	0.0163^*^	0.0163^*^
	(1.946)	(1.939)	(1.934)	(1.942)	(1.942)
*INSTITU*	−0.0167^***^	−0.0167^***^	−0.0166^***^	−0.0166^***^	−0.0166^***^
	(−3.278)	(−3.275)	(−3.263)	(−3.268)	(−3.267)
*STATE*	−0.0027	−0.0026	−0.0026	−0.0026	−0.0027
	(−1.095)	(−1.093)	(−1.091)	(−1.091)	(−1.093)
*Intercept*	0.0772^***^	0.0769^***^	0.0778^***^	0.0767^***^	0.0762^***^
	(6.820)	(6.783)	(6.825)	(6.703)	(6.622)
Industry	Yes	Yes	Yes	Yes	Yes
Year	Yes	Yes	Yes	Yes	Yes
*N*	5,980	5,980	5,980	5,980	5,980
*R^2^*	0.1210	0.1209	0.1211	0.1208	0.1208

All standard errors are clustered by firm. The t-statistics are reported in parentheses below the coefficient estimates.

***, **, and * represent statistically significant at the 1, 5, and 10% levels, respectively.

This table conducts the pseudo–year policy robustness check in our study for only Jones' model. Specification (1) RIGHT is in exactly the year 2016. Specification (2) −1 is 1 year before. Specification (3) −2 is 2 years earlier. Specification (4) −3 is 3 years earlier. Specification (5) −4 is 4 years earlier. Coefficients measure the effect of social insurance contributions burden on the discretionary accruals for different groups. Our variable of interest is social insurance contributions burden SIC, which is the ratio of social insurance contributions over revenue. All specifications include year and industrial dummy, which are omitted in this table.

Source: The author's calculations are based on data from the sources indicated in Section Sample and data collection.

**Figure 3:**
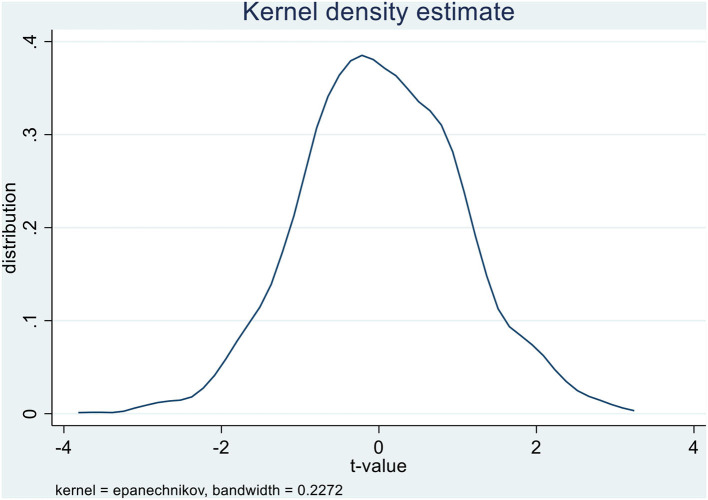
Placebo test −1,000 times distribution. This figure shows the placebo test distribution for 1,000 times regression results. It plots the *t*-value for the variable *POLICY_TREAT* around zero. Source: The author's calculations are based on data from the sources indicated in Section Sample and data collection.

To investigate whether the effect of the previous policy is interfered with by other non-observed random factors, this paper uses the method of non-participation replacement test to construct a placebo test based on Chetty et al. (2009). The procedure is as follows: First, we randomly draw the sample with the same number of observations as the real treated groups and the other firms are used as the control group; Second, we set the interaction term *POLICY_TREAT* based on the randomly selected firms of the treated group and the year in which the social insurance policy was implemented; Third, we keep the other control variables unchanged and substitute the interaction terms into the previous model for regression analysis. Figure 3 describes the probability distribution of the estimated coefficients of the interaction terms under 1,000 times random sampling. It can be found from Figure 3 that the estimated coefficient value obtained from random sampling is distributed around 0.

## Further analysis

Previously, we have shown that the social insurance contributions burden significantly reduces the degree of earnings management. One possible explanation is that the social insurance contributions burden affects earnings management by reducing the level of free cash flow. Next, this paper will further explore the impact on earnings management from the perspective of labor cost in general. In addition to the internal and external social security pressures such as labor density and pension looseness mentioned earlier, this paper will also explore the circumstances under which enterprises are more inclined to reduce earnings management when facing a higher social insurance contributions burden. The factors such as financing constraints and the impact of the external financing environment are being considered. Besides, this paper tries to reveal whether earnings management is a short-term behavior or a long-term strategy in correlation with the social insurance contributions burden, and what impact earnings management will have on enterprise value and market performance. Finally, given the positive impact of earnings management caused by the social insurance contributions burden, this paper examines whether the internal and external corporate governance mechanism can play a governance role.

### Employee payroll and earnings management

Social insurance is an important part of the employee payroll system, and it is mandatory. Next, this paper tries to explore whether the effects of employee compensation on earnings management are the same as the social insurance contributions burden from a broader perspective. In other words, this paper empirically tests whether employee compensation reduces the degree of earnings management to further clarify why the social insurance contributions burden reduces the level of earnings management. According to the extant literature, this paper uses two methods to measure the labor cost of an enterprise: one is the ratio of cash paid for employees to revenue (*LABOR*); the other is the log of average salary per employee (*WAGE*), which is the log of the total payroll payable to employees divided by the number of employees. Table 10 shows that *LABOR* significantly reduces earnings management at the 1% level, while *WAGE* insignificantly increases earnings management. This implies that the cash and social insurance contributions paid to employees reduced the level of earnings management, while the payroll payable to employees' accounts is much easier to manipulate. To some extent, the payroll payable to employees' accounts increases the room for manipulation, thus increasing the level of earnings management. The conclusion in this part is somehow inconsistent with previous literature, and it deserves further investigation in the future.

**Table 10:** Labor cost and earnings management.

	**(1)**	**(2)**	**(3)**	**(4)**
	***DA***	***MODDA***	***DA***	***MODDA***
*LABOR*	−0.0490^***^	−0.0515^***^		
	(−4.411)	(−4.543)		
*SIZE*	−0.0064^***^	−0.0066^***^	−0.0058^***^	−0.0059^***^
	(−8.071)	(−8.155)	(−7.024)	(−7.014)
*LEV*	0.0621^***^	0.0628^***^	0.0654^***^	0.0664^***^
	(12.526)	(12.350)	(13.316)	(13.142)
*ROA*	0.2998^***^	0.3067^***^	0.3007^***^	0.3084^***^
	(13.733)	(13.630)	(13.955)	(13.813)
*CAPITAL*	−0.0550^***^	−0.0558^***^	−0.0565^***^	−0.0577^***^
	(−10.570)	(−10.577)	(−10.734)	(−10.816)
*FIRST*	0.0094^*^	0.0101^**^	0.0091^*^	0.0101^*^
	(1.904)	(1.990)	(1.822)	(1.956)
*INSTITU*	−0.0120^***^	−0.0127^***^	−0.0123^***^	−0.0132^***^
	(−3.759)	(−3.884)	(−3.869)	(−4.035)
*STATE*	−0.0038^**^	−0.0038^**^	−0.0046^***^	−0.0047^***^
	(−2.384)	(−2.325)	(−2.896)	(−2.825)
*WAGE*			0.0018	0.0018
			(1.232)	(1.142)
*Intercept*	0.2179^***^	0.2289^***^	0.1832^***^	0.1935^***^
	(12.157)	(12.585)	(8.432)	(8.732)
Industry	Yes	Yes	Yes	Yes
Year	Yes	Yes	Yes	Yes
*N*	18,224	18,224	18,055	18,055
*R^2^*	0.1291	0.1276	0.1251	0.1236

All standard errors are clustered by firm. The t-statistics are reported in parentheses below the coefficient estimates.

***, **, and * represent statistically significant at the 1, 5, and 10% levels, respectively.

This table presents the regression results in our study for both Jones' and modified Jones' models. Specifications (1) DA_SOE and (2) MODDA are for the labor cost group. Specifications (3) DA and (4) MODDA are for the average wage group. Coefficients measure the effect of social insurance contributions burden on the discretionary accruals for different groups. Our variable of interest is social insurance contributions burden SIC, which is the ratio of social insurance contributions over revenue. All specifications include year and industrial dummy, which are omitted in this table.

Source: The author's calculations are based on data from the sources indicated in Section Sample and data collection.

### Financing constraints and external financing environment

The financing constraints faced by firms and the external financing environment may also have an important influence on the relationship between social insurance contributions burden and earnings management. It is expected that if the firms' financing constraints are tight when facing the social insurance contributions burden, mangers have fewer opportunities for earnings management. On the other hand, if the external marketization of enterprises in their regions is lower and the financial markets are less developed, it is more difficult for firms to obtain financial support from outsiders when facing the social insurance contributions burden, so the firms have more motivated to manipulate in order to make the financial statement attractive, resulting in higher earnings management. This paper uses the *KZ-index* (Kaplan and Zingales, 1997). The *KZ-index* measures the degree of financing constraints faced by a single listed firm; the greater the *KZ-index* value, the higher the degree of financing constraints. This paper also uses the financial marketization index to measure the external financing environment of the provincial regions in which the listed firms are located; the greater the financial marketization index value, the higher the degree of financial development and the better the external financing environment. Table 11 Panel A shows that the negative relation between social insurance contributions burden (*SIC*) and earnings management is more pronounced in the group with high financing constraints. Chow's test (Chow, 1960) is statistically significant at the 1% level. It verifies that the difference in coefficients between sub-samples is statistically significant. When the degree of financing constraints is higher, the listed firms will face a heavier and greater social insurance contribution burden. Then, the money to spend for managers is limited and there are fewer opportunities for earnings management. However, the regression results in Panel B of Table 11 show that the social insurance contributions burden (*SIC*) significantly decreases the degree of earnings management in the low financial marketization group. Additionally, Chow's test is statistically significant at the 1% level. It verifies that the difference in coefficients between sub-samples is statistically significant. When the external financing environment is poor, the firms are more likely to alleviate external financing difficulties through earnings management under the pressure of the social insurance contributions burden.

**Table 11:** Financing constraints and external financing environment.

	**A: Financing constraint**		**B: Marketization**	
	**Low**	**High**	**Low**	**High**
	**(1)**	**(2)**	**(1)**	**(2)**
	***DA***	***DA***	***DA***	***DA***
*SIC*	−0.1835^*^	−0.1981^**^	−0.2429^***^	−0.2102^**^
	(−1.664)	(−2.350)	(−2.731)	(−2.248)
*CONTROLS*	Yes	Yes	Yes	Yes
Industry	Yes	Yes	Yes	Yes
Year	Yes	Yes	Yes	Yes
*Intercept*	0.2182^***^	0.2011^***^	0.2041^***^	0.2343^***^
	(6.831)	(9.300)	(7.452)	(9.853)
Chow's test	0.0000^***^		0.0028^***^
*N*	8,724	9,460	8,227	9,999
*R^2^*	0.1277	0.1583	0.1273	0.1208

All standard errors are clustered by firm. The t-statistics are reported in parentheses below the coefficient estimates.

***, **, and * represent statistically significant at the 1, 5, and 10% levels, respectively.

This table presents the regression results in our study for Jones' model. Specification (1) DA is for the low financing constraint group. Specification (2) DA is for a high financing constraint enterprise group. Specification (3) DA is for low marketization regional enterprise groups. Specification (4) DA is for high marketization regional enterprise group. Coefficients measure the effect of social insurance contributions burden on the discretionary accruals for different groups. Our variable of interest is social insurance contributions burden SIC, which is the ratio of social insurance contributions over revenue. All specifications include year and industrial dummy, which are omitted in this table.

Source: The author's calculations are based on data from the sources indicated in Section Sample and data collection.

### The internal and external corporate governance mechanisms

The internal and external corporate governance mechanisms play an effective role in the earnings management process under the social insurance contributions burden. For the internal corporate governance mechanism, this paper selects the proportion of management shareholding as a measure to reflect the characteristics of internal corporate governance. A higher proportion of management shareholding implies a closer tie between the incentive of shareholders and managers, and the agency cost is lower, so the internal corporate governance is better. As shown in Table 12, the social insurance contributions burden (*SIC*) has a greater negative effect on earnings management in the high proportion of management shareholding. And Chow's test is statistically significant at the 1% level. It verifies that the difference in coefficients between sub-samples is statistically significant. This means that when managers have a higher shareholding ratio and internal corporate governance is relatively better, the pressure on social insurance contributions burden increases which may cause the degree of earnings management to drop even further.

**Table 12:** Internal corporate governance.

	**Panel A: Internal corporate governance**	
	**Low**	**High**
	**(1)**	**(2)**
	***DA***	***DA***
*SIC*	−0.1966^**^	−0.2192^**^
	(−2.026)	(−2.509)
*CONTROLS*	Yes	Yes
Industry	Yes	Yes
Year	Yes	Yes
*Intercept*	0.2118^***^	0.1959^***^
	(8.628)	(7.868)
Chow's test	0.0044^***^
*N*	8,507	9,677
*R^2^*	0.1335	0.1295

All standard errors are clustered by firm. The t–statistics are reported in parentheses below the coefficient estimates.

***, **, and * represent statistically significant at the 1, 5, and 10% levels, respectively.

This table presents the regression results in our study for Jones' model. Specification (1) DA is for a low internal corporate governance group. Specification (2) DA is for an internal corporate governance enterprise group. Coefficients measure the effect of social insurance contributions burden on the discretionary accruals for different groups. Our variable of interest is social insurance contributions burden SIC, which is the ratio of social insurance contributions over revenue. All specifications include year and industrial dummy, which are omitted in this table.

Source: The author's calculations are based on data from the sources indicated in Section Sample and data collection.

For the external corporate governance mechanism in Table 13, we explore the different social insurance contributions collection systems in China. From 2008 to 2017, the social insurance contributions were collected in three ways across different regions. Beijing, Shanghai, Tianjin, Shenzhen, Shanxi, Jilin, Jiangxi, Shandong, Guangxi, Sichuan, Guizhou, Tibet, and Xinjiang are all collected by social security agencies. Liaoning, Zhejiang, Fujian, Guangdong, and Xiamen are collected by the local tax bureau with full responsibility. The rest are collected on behalf of tax authorities. Specifically, we test how these different means of collecting systems influence the results of social insurance contributions burden on the earnings management. We first regress based on the collecting agencies. The results show that the tax bureau is not an effective governance mechanism, while the social security agency acting effectively. The rationale behind this is that the tax bureau might have an incentive to collect more tax rather than social insurance contributions. However, when we categorize *via* the collecting system in Table 14, the results show that the negative impact of social insurance contributions burden (*SIC*) on the earnings management is most significant by the local tax bureau with a full responsibility group. Chow's test is statistically significant at the 1% level. It verifies that the difference in coefficients among sub-samples is statistically significant. This is reasonable since the tax bureau has more accessible information and greater power to improve the collecting rate. The social insurance collected *via* the social security agency group has a relatively moderate effect. On the contrary, the social insurance contributions burden does not have a significant effect on the earnings management if the social insurance is collected on behalf of tax authorities since there might be a trade-off between tax and social insurance collecting. This suggests that the social insurance contributions collected by the local tax bureau with full responsibility are the most effective external corporate governance mechanism. This paper concludes that good internal and external corporate governance mechanisms such as management shareholding and collecting systems can effectively restrain the earnings management behavior under the social insurance contributions burden.

**Table 13:** External corporate governance categorized by collecting agencies.

	**(1)**	**(2)**	**(3)**	**(4)**
	***DA_TAX***	***DA_SIC***	***MODDA_TAX***	***MODDA_SIC***
*SIC*	−0.1242	−0.2212^**^	−0.1458	−0.2227^**^
	(−1.379)	(−2.421)	(−1.587)	(−2.357)
*LEV*	0.0515^***^	0.0550^***^	0.0522^***^	0.0545^***^
	(8.225)	(8.389)	(8.089)	(8.064)
*ROA*	0.2740^***^	0.2911^***^	0.2860^***^	0.2896^***^
	(9.330)	(8.795)	(9.416)	(8.446)
*CAPITAL*	−0.0486^***^	−0.0534^***^	−0.0492^***^	−0.0537^***^
	(−7.511)	(−7.130)	(−7.383)	(−7.142)
*FIRST*	0.0096	0.0061	0.0105	0.0076
	(1.508)	(0.817)	(1.591)	(0.989)
*INSTITU*	−0.0185^***^	−0.0159^***^	−0.0201^***^	−0.0163^***^
	(−4.565)	(−3.291)	(−4.780)	(−3.266)
*STATE*	−0.0037^*^	−0.0072^***^	−0.0029	−0.0081^***^
	(−1.765)	(−2.874)	(−1.340)	(−3.140)
*Intercept*	0.0765^***^	0.0836^***^	0.0817^***^	0.0897^***^
	(8.723)	(9.182)	(9.128)	(9.335)
Industry	Yes	Yes	Yes	Yes
Year	Yes	Yes	Yes	Yes
*N*	10185	7999	10185	7999
*R^2^*	0.1080	0.1310	0.1086	0.1284

All standard errors are clustered by firm. The t–statistics are reported in parentheses below the coefficient estimates.

***, **, and * represent statistically significant at the 1, 5, and 10% levels, respectively.

This table presents the regression results in our study for both Jones' and modified Jones' models. Specifications (1) DA_TAX and (3) MODDA_TA are for tax bureau as collecting agencies group. Specifications (2) DA_SIC and (4) are for social security administration as a collecting agencies group. Coefficients measure the effect of social insurance contributions burden on the discretionary accruals for different groups. Our variable of interest is social insurance contributions burden SIC, which is the ratio of social insurance contributions over revenue. All specifications include year and industrial dummy, which are omitted in this table.

Source: The author's calculations are based on data from the sources indicated in Section Sample and data collection.

**Table 14:** External corporate governance categorized by collecting systems.

	**(1)**	**(2)**	**(3)**
	***DA_TAX***	***DA_***	***DA_***
	***AGENT***	***SIC***	***TAXFULL***
*SIC*	−0.0170	−0.2212^**^	−0.4657^***^
	(−0.155)	(−2.421)	(−3.079)
*LEV*	0.0455^***^	0.0550^***^	0.0585^***^
	(5.686)	(8.389)	(5.941)
*ROA*	0.2584^***^	0.2911^***^	0.2841^***^
	(7.061)	(8.795)	(6.108)
*CAPITAL*	−0.0340^***^	−0.0534^***^	−0.0637^***^
	(−4.031)	(−7.130)	(−6.219)
*FIRST*	−0.0019	0.0061	0.0228^**^
	(−0.243)	(0.817)	(2.192)
*INSTITU*	−0.0192^***^	−0.0159^***^	−0.0185^***^
	(−3.677)	(−3.291)	(−2.850)
*STATE*	−0.0027	−0.0072^***^	−0.0076^**^
	(−1.035)	(−2.874)	(−2.138)
*Intercept*	0.0773^***^	0.0836^***^	0.0797^***^
	(7.148)	(9.182)	(5.926)
Chow's test	0.0012^***^
Industry	Yes	Yes	Yes
Year	Yes	Yes	Yes
*N*	5,780	7,999	4,405
*R^2^*	0.0919	0.1310	0.1420

All standard errors are clustered by firm. The t-statistics are reported in parentheses below the coefficient estimates.

***, **, and * represent statistically significant at the 1, 5, and 10% levels, respectively.

This table presents the regression results in our study for Jones' model. Specification (1) DA_TAXAGENT is for the local tax bureau acting as a collecting agent group. Specification (2) DA_SIC is for social security administration as the collecting group. Specification (3) DA_TAXFULL is for the local tax bureau with a full collecting responsibility group. Coefficients measure the effect of social insurance contributions burden on the discretionary accruals for different groups. Our variable of interest is social insurance contributions burden SIC, which is the ratio of social insurance contributions over revenue. All specifications include year and industrial dummy, which are omitted in this table.

Source: The author's calculations are based on data from the sources indicated in Section Sample and data collection.

### Tax avoidance and social insurance contributions burden

A natural continuation from the previous test is whether those firms that avoid taxes are more likely to avoid social insurance contributions as well. Thus, we further partition these firms into two groups, one with high tax avoidance and the other with low tax avoidance. We run the regression based on the collecting system. From Table 15, we find that the social insurance contributions burden plays an effective role for firms with low tax avoidance, but it is not significant for high tax avoidance groups regardless of the collecting systems. Chow's test is statistically significant at the 10% level. It verifies that the difference in coefficients between sub-samples is statistically significant. Further examination shows that there might be a trade-off between paying taxes and making contributions to social insurance for the collecting system on behalf of the tax bureau, and the tax bureau collecting system with full responsibility plays the most effective governance role in terms of earnings management.

**Table 15:** Tax avoidance and social insurance contributions burden.

	**(1)**	**(2)**	**(3)**	**(4)**	**(5)**	**(6)**
	***DA1_***	***DA2_***	***DA1_***	***DA2_***	***DA1_***	***DA2_***
	***TAXAGENT***	***TAXAGENT***	***SIC***	***SIC***	***TAXFULL***	***TAXFULL***
*SIC*	−0.2848^**^	0.3111	−0.4355^***^	−0.0572	−0.6868^***^	−0.3022
	(−2.305)	(1.594)	(−3.573)	(−0.419)	(−3.492)	(−1.393)
*SIZE*	−0.0069^***^	−0.0054^**^	−0.0063^***^	−0.0046^***^	−0.0058^***^	−0.0054^**^
	(−3.744)	(−2.522)	(−4.125)	(−2.852)	(−2.751)	(−2.426)
*LEV*	0.0429^***^	0.0679^***^	0.0561^***^	0.0734^***^	0.0615^***^	0.0742^***^
	(4.459)	(4.374)	(5.819)	(6.957)	(4.910)	(4.450)
*ROA*	0.3091^***^	0.3443^***^	0.2934^***^	0.4491^***^	0.3352^***^	0.3607^***^
	(3.937)	(6.115)	(4.549)	(9.217)	(3.632)	(5.514)
*CAPITAL*	−0.0363^***^	−0.0478^***^	−0.0484^***^	−0.0780^***^	−0.0440^***^	−0.0883^***^
	(−3.388)	(−3.688)	(−3.983)	(−7.263)	(−3.429)	(−5.980)
*FIRST*	0.0071	−0.0127	−0.0029	0.0175^*^	0.0351^***^	0.0210
	(0.724)	(−1.024)	(−0.257)	(1.675)	(2.661)	(1.492)
*INSTITU*	−0.0078	−0.0197^**^	−0.0029	−0.0122^*^	−0.0321^***^	−0.0027
	(−1.121)	(−2.510)	(−0.407)	(−1.791)	(−3.355)	(−0.303)
*STATE*	−0.0027	−0.0040	−0.0030	−0.0065^*^	−0.0051	−0.0039
	(−0.828)	(−1.022)	(−0.902)	(−1.752)	(−0.965)	(−0.741)
*Intercept*	0.2299^***^	0.2148^***^	0.2390^***^	0.1747^***^	0.1475^***^	0.1786^***^
	(5.928)	(3.844)	(6.459)	(5.043)	(3.455)	(3.936)
Chow's test	0.0979^*^		0.0979^*^		0.0979^*^	
Industry	Yes	Yes	Yes	Yes	Yes	Yes
Year	Yes	Yes	Yes	Yes	Yes	Yes
*N*	3,001	2,779	3,904	4,095	2,070	2,335
*R^2^*	0.1079	0.1282	0.1607	0.1412	0.1789	0.1714

All standard errors are clustered by firm. The t-statistics are reported in parentheses below the coefficient estimates.

***, **, and * represent statistically significant at the 1, 5, and 10% levels, respectively.

This table presents the regression results in our study for only Jones' model. Specifications (1) DA1_TAXAGENT and (2) DA2_TAXAGENT are for low and high tax avoidance enterprises with tax bureau as collecting agent groups respectively. Specifications (3) DA1_SIC and (4) DA1_SIC are for low and high tax avoidance enterprises with social security administration as collector groups, respectively. Specifications (5) DA1_TAXFULL and (6) DA2_TAXFULL are for low and low tax avoidance enterprises with full collecting responsibility groups, respectively. Our variable of interest is social insurance contributions burden SIC, which is the ratio of social insurance contributions over revenue. All specifications include year and industrial dummy, which are omitted in this table.

Source: The author's calculations are based on data from the sources indicated in Section Sample and data collection.

## Conclusion

For a long time, government officials and academics in China have been deeply concerned about the burden of social insurance contributions. However, there is a limited amount of literature to explore the impact of social insurance contributions on financial behavior from the micro perspective. This problem has great theoretical and practical significance under the ongoing policy reform of tax and fee reduction. Based on the research sample of A-share listed companies in China from 2008 to 2017, this paper empirically tests the relationship between social insurance contributions burden and earnings management and its mediating mechanism, examines the role of internal and external social security pressure in the relationship between the two, and tries to understand the internal logic of social insurance policy. This paper finds that the social insurance contributions burden significantly reduces earnings management. The mechanism behind this is that the social insurance contributions burden reduces the level of free cash flow, resulting in fewer over-investments and over-consumption from the managers, thus reducing the agency cost of the firms, and subsequently decreasing the degree of earnings management. At the same time, the negative impact of social insurance contributions burden on earnings management is particularly pronounced when the internal and external social insurance pressure is high. Further research also shows that different measures of labor costs give mixed results in earnings management. The negative relation between social insurance contributions burden and earnings management is more pronounced in areas with a higher degree of financing constraints and a lower degree of financial marketization. Finally, an effective internal corporate governance mechanism can help to restrain earnings management under the pressure of social insurance contributions burden, while the effects of the external corporate governance mechanism need to reconsider.

For policy makers, this paper suggests that blindly reducing the social insurance rate will not only reduce the welfare of corporate employees but also create room for corporate governance. This paper finds that even if the social insurance contributions burden is reduced, because of the increase in agency cost, managers are more likely to over-invest and waste excess free cash flow, causing more severe earnings management problems. This means that social insurance rate cuts may have a negative corporate governance effect. The policymakers should, therefore, reasonably control the rate rather than keep reducing it based on the actual situation of firms. It is necessary to effectively stimulate the vitality of firms and promote high-quality economic development. Further analysis shows that although the social insurance administration function as a more effective collecting agency, the local tax bureau with full responsibility plays the most effective governance role relative to other collecting methods. Thus, we suggest making the social insurance agency a subdivision of the local tax bureau and taking full responsibility as the optimal social insurance collecting system, and for shareholders, reasonable control of managers for the use of free cash flow is the most important thing. Firms should put more effort into corporate governance to effectively prevent over-investment, over-consumption, transfer of benefits, and other misconduct behaviors. Firms should construct long-term development strategies and take effective measures to deal with the opportunities and challenges brought by the social insurance contributions burden. This paper finds that social security benefits and good corporate governance complement each other. Therefore, firms should take effective measures to improve the efficiency of corporate governance and not by reducing the social insurance contributions burden. The conclusion of this paper provides an alternative view of the ongoing reform policy of reducing the social insurance rate in China. The paper suggests that helping firms reduce the social insurance burden is not necessarily the goal. Social insurance contributions burden is not just a burden, but also a restriction measure to managers in which both employees and shareholders benefit.

## Data availability statement

Both CSMAR and WIND datasets require membership and requests to access these datasets should be directed to dataservice@csmar.com / https://www.wind.com.cn/. Any further inquiries can be directed to the corresponding author.

## Author contributions

YB contributed to funding, ideas, and writing. BZ was responsible for data analysis. Both authors contributed to the article and approved the submitted version.

## Funding

We appreciate financial support by National Natural Science Foundation of China (NSFC) [Grant No. 72272108] for providing the funding for this study.

## Conflict of interest

The authors declare that the research was conducted in the absence of any commercial or financial relationships that could be construed as a potential conflict of interest.

## Publisher's note

All claims expressed in this article are solely those of the authors and do not necessarily represent those of their affiliated organizations, or those of the publisher, the editors and the reviewers. Any product that may be evaluated in this article, or claim that may be made by its manufacturer, is not guaranteed or endorsed by the publisher.
